# The European Bioinformatics Institute: empowering cooperation in response to a global health crisis

**DOI:** 10.1093/nar/gkaa1077

**Published:** 2020-11-27

**Authors:** Gaia Cantelli, Guy Cochrane, Cath Brooksbank, Ellen McDonagh, Paul Flicek, Johanna McEntyre, Ewan Birney, Rolf Apweiler

**Affiliations:** European Molecular Biology Laboratory, European Bioinformatics Institute (EMBL-EBI), Wellcome Genome Campus, Hinxton, Cambridge CB10 1SD, UK; European Molecular Biology Laboratory, European Bioinformatics Institute (EMBL-EBI), Wellcome Genome Campus, Hinxton, Cambridge CB10 1SD, UK; European Molecular Biology Laboratory, European Bioinformatics Institute (EMBL-EBI), Wellcome Genome Campus, Hinxton, Cambridge CB10 1SD, UK; European Molecular Biology Laboratory, European Bioinformatics Institute (EMBL-EBI), Wellcome Genome Campus, Hinxton, Cambridge CB10 1SD, UK; Open Targets, European Bioinformatics Institute (EMBL-EBI), Wellcome Genome Campus, Hinxton, Cambridgeshire CB10 1SD, UK; European Molecular Biology Laboratory, European Bioinformatics Institute (EMBL-EBI), Wellcome Genome Campus, Hinxton, Cambridge CB10 1SD, UK; European Molecular Biology Laboratory, European Bioinformatics Institute (EMBL-EBI), Wellcome Genome Campus, Hinxton, Cambridge CB10 1SD, UK; European Molecular Biology Laboratory, European Bioinformatics Institute (EMBL-EBI), Wellcome Genome Campus, Hinxton, Cambridge CB10 1SD, UK; European Molecular Biology Laboratory, European Bioinformatics Institute (EMBL-EBI), Wellcome Genome Campus, Hinxton, Cambridge CB10 1SD, UK

## Abstract

The European Bioinformatics Institute (EMBL-EBI; https://www.ebi.ac.uk/) provides freely available data and bioinformatics services to the scientific community, alongside its research activity and training provision. The 2020 COVID-19 pandemic has brought to the forefront a need for the scientific community to work even more cooperatively to effectively tackle a global health crisis. EMBL-EBI has been able to build on its position to contribute to the fight against COVID-19 in a number of ways. Firstly, EMBL-EBI has used its infrastructure, expertise and network of international collaborations to help build the European COVID-19 Data Platform (https://www.covid19dataportal.org/), which brings together COVID-19 biomolecular data and connects it to researchers, clinicians and public health professionals. By September 2020, the COVID-19 Data Platform has integrated in excess of 170 000 COVID-19 biomolecular data and literature records, collected through a number of EMBL-EBI resources. Secondly, EMBL-EBI has strived to continue its support of the life science communities through the crisis, with updated Training provision and improved service provision throughout its resources. The COVID-19 pandemic has highlighted the importance of EMBL-EBI’s core principles, including international cooperation, resource sharing and central data brokering, and has further empowered scientific cooperation.

## INTRODUCTION

**Figure 1.**
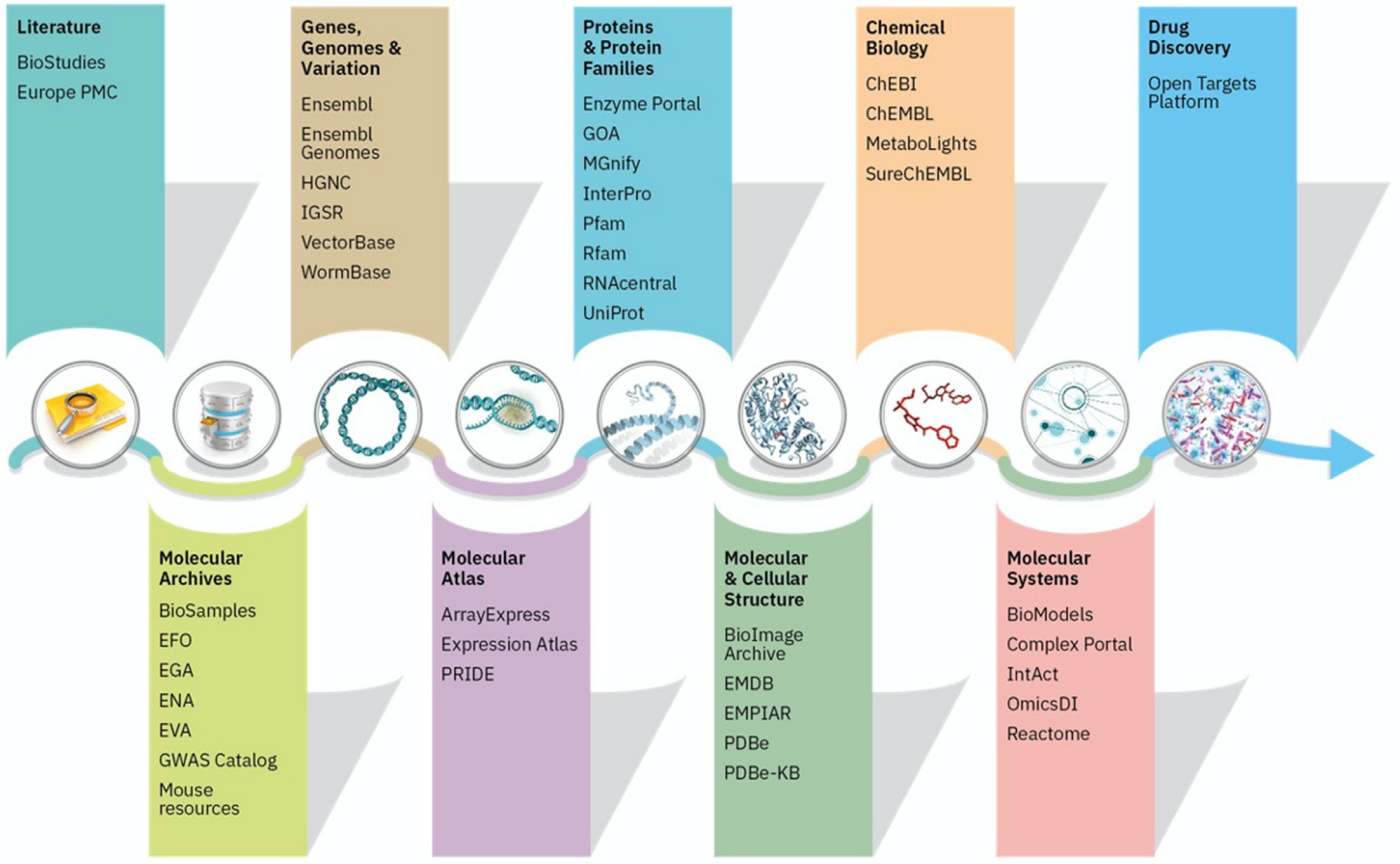
EMBL-EBI Resources. Summary of all EMBL-EBI Data Resources as of September 2020.

The European Bioinformatics Institute (EMBL-EBI) is part of the European Molecular Biology Laboratory, an intergovernmental research organisation focusing on advancing the study and understanding of molecular biology across Europe. EMBL-EBI provides freely available data and bioinformatics services to the scientific community, contributing to the advancement of biology through investigator-driven research and supporting scientists at all levels with advanced bioinformatics training. We host a suite of open data resources and tools, which cover every data type in molecular biology, including, among others, nucleotide sequence data, protein sequences, chemical biology and the scientific literature (Figure 1). Resources hosted by EMBL-EBI include deposition databases (which archive experimental data) and added-value databases (which add value to archived data by providing annotation, curation, reanalysis and integration), as well as open-source software tools. EMBL-EBI resources are central to the life sciences community. On an average day in 2019, EMBL-EBI resources received over 62 million requests. Throughout 2019, EMBL-EBI websites were visited by almost 24 million IP addresses (the number of IP addresses is an indication of the number of users, but not an exact count), and EMBL-EBI trained over 30 000 people through its training programmes. Moreover, in 2019 EMBL-EBI reached over 300 petabytes of raw data storage space.

EMBL-EBI is committed to keeping the data it manages open, freely accessible and FAIR (findable, accessible, interoperable and reusable (1)). Building on its 25 years of international collaboration in bioinformatics and data brokering, EMBL-EBI is working with the international scientific community to build new scientific partnerships and coordinate global research efforts. This involves, for example, active participation in the development of the Global Alliance for Genomics and Health (https://www.ga4gh.org/), leading in the creation of standardised methods for accessing large-scale genomic data, and the use of the Data Use Ontology (2) by the European Genome-phenome Archive (EGA) to ensure that data use conditions are machine readable and standardised. Moreover, EMBL-EBI is a key player in the ARGENT project, an international effort that aims to create a global, live database of *Mycobacterium tuberculosis* genomes to monitor tuberculosis outbreaks around the world. Through these efforts, EMBL-EBI is developing new ways for researchers, clinicians and public health professionals to share data, gather new insights and build on each other's discoveries.

The 2020 COVID-19 pandemic has re-shaped the landscape of research and scientific cooperation. The Severe Acute Respiratory Syndrome Coronavirus 2 (SARS-CoV-2) causes the disease COVID-19: between January and September 2020, SARS-CoV-2 has been responsible for over 890 000 deaths. Moreover, the pandemic and the lockdown measures implemented by governments across the globe to contain it have caused unprecedented economic and societal disruption. The pandemic showed that the scientific community needed effective international collaboration, backed by trusted expertise and an open dialogue with the public. The position EMBL-EBI holds as an international data broker, a service provider and home to cutting-edge research has allowed the Institute to contribute to the fight against COVID-19 in a number of ways. Crucially, the contributions of the Institute hinge not solely on coronavirus research, but on empowering international cooperation across research fields, allowing researchers to access trustworthy information and to cooperate in the face of a global crisis.

## DATA SERVICE RESPONSES TO THE PANDEMIC

**Figure 2.**
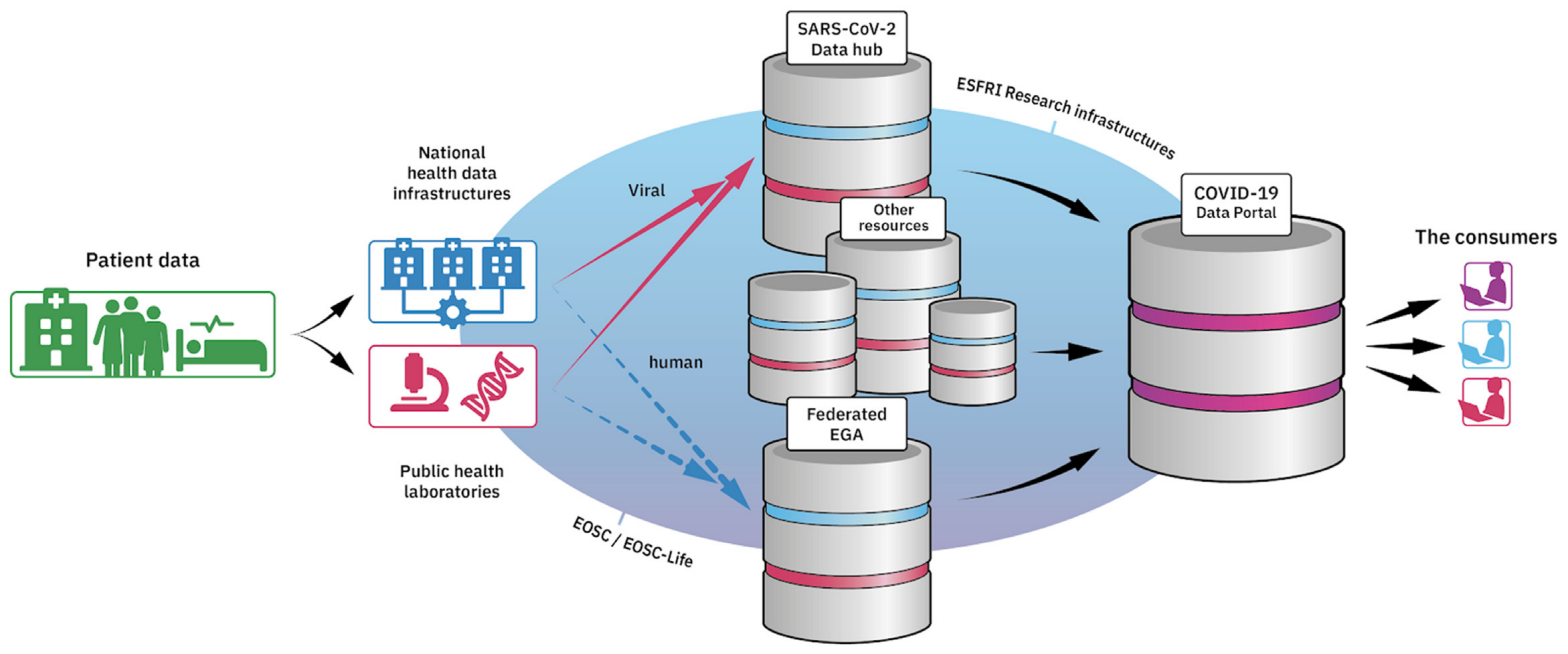
Data flow through the components of the European COVID-19 Data Platform. Patient data is collected by national health data infrastructures and public health laboratories, which can be deposited in specific SARS-CoV-2 Data Hubs (numbering 12 at the time of writing), Federated EGA (FEGA) and other data resources. This data feed into the COVID-19 Data Portal, which can be accessed by its user community of researchers, practitioners and public health specialists.

In the early months of 2020, the future global impact of the newly emerged SARS-CoV-2 became apparent. From March 2020, EMBL-EBI, supported by the European Commission and other funders, set to work on building a foundation for the scientific response to COVID-19 centred on biomolecular data. On the 20th of April 2020 the **European COVID-19 Data Platform** (https://www.covid19dataportal.org/) was launched, as announced by the President of the European Commission (https://audiovisual.ec.europa.eu/en/video/I-189639). The platform enables researchers to upload, access and analyse COVID-19 related reference data and specialist datasets as part of the global research effort. Drawing on our infrastructure, membership of ELIXIR and involvement in the European Open Science Cloud, we developed resources to link the clinical, epidemiological, and public health worlds. The European COVID-19 Data Platform comprises three components: the COVID-19 Data Portal, the SARS-CoV-2 Data Hub and the Federated EGA (FEGA) (Figure 2).

### The COVID-19 Data Portal

**Figure 3.**
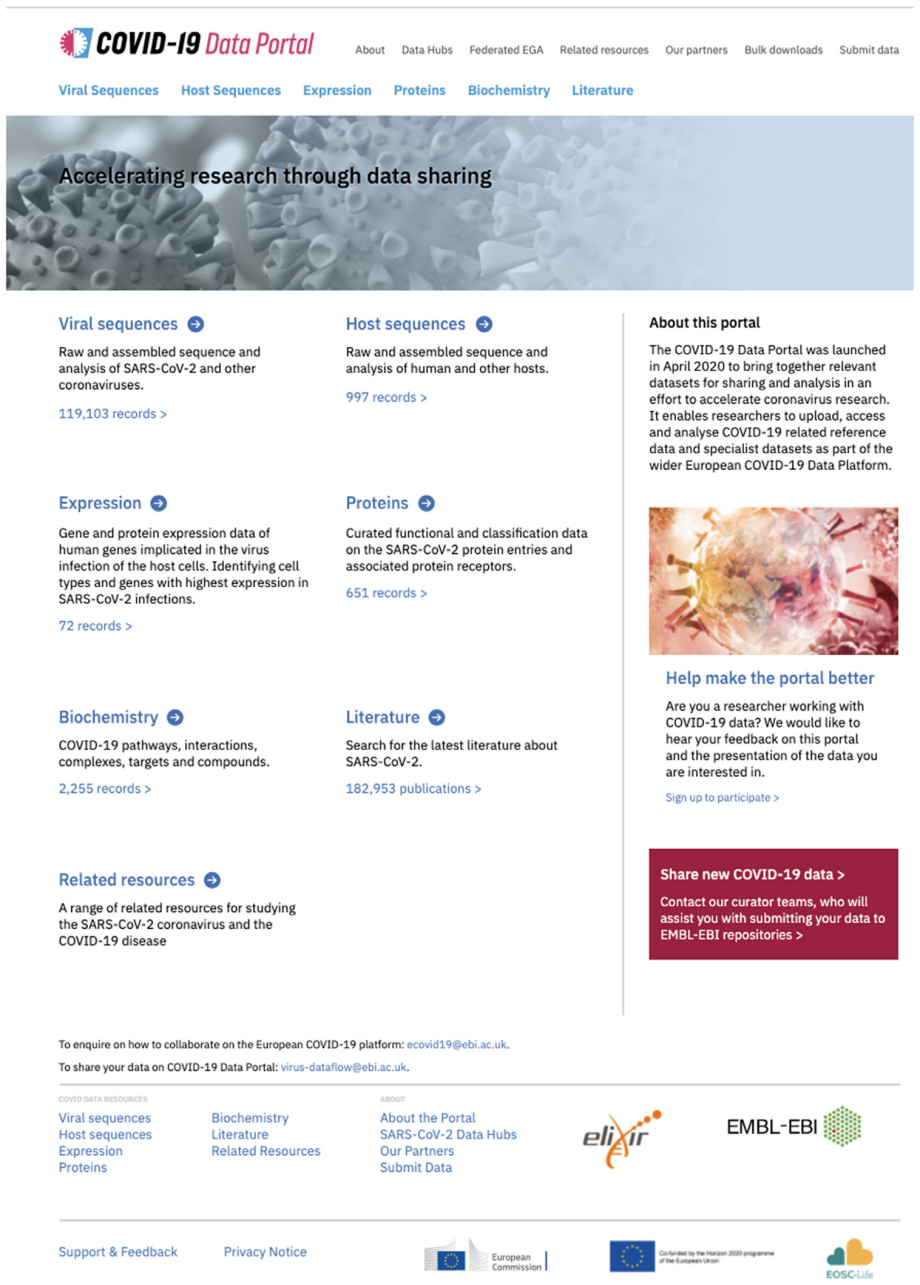
The COVID-19 Data Portal interface. The COVID-19 Data Portal provides a web entry point to services provided by the European COVID-19 Data Platform.

The COVID-19 Data Portal (https://www.covid19dataportal.org, Figure 3) brings together biomolecular data across the diversity of relevant data types to provide an integrated search (based on EMBL-EBI’s central data search service (3)), navigation across data and data download. In addition, the Portal provides an entry point to data resources and services beyond the biomolecular domain, including clinical and epidemiology data.

In September 2020, the system presents data from 14 of EMBL-EBI’s biomolecular data resources, including UniProt (4), European Nucleotide Archive (5), Electron Microscopy Data Bank (5), Protein Data Bank in Europe (6), Europe PMC (7), PRIDE (8), InterPro (9), ChEMBL (10), Complex Portal (11), Protein Data Bank in Europe-Knowledge Base (12), Ensembl (13), Reactome (14) IntAct (15) and the European Genome-phenome Archive (16), featuring many of ELIXIR’s Core Data Resources (17). Data in the system is growing rapidly and spans viral and host sequences, gene and protein expression, protein functions and structures, biochemical pathways and compounds, and the scientific literature (Box 1).

**Table:** Data content available from the COVID-19 Data Portal (September 2020).

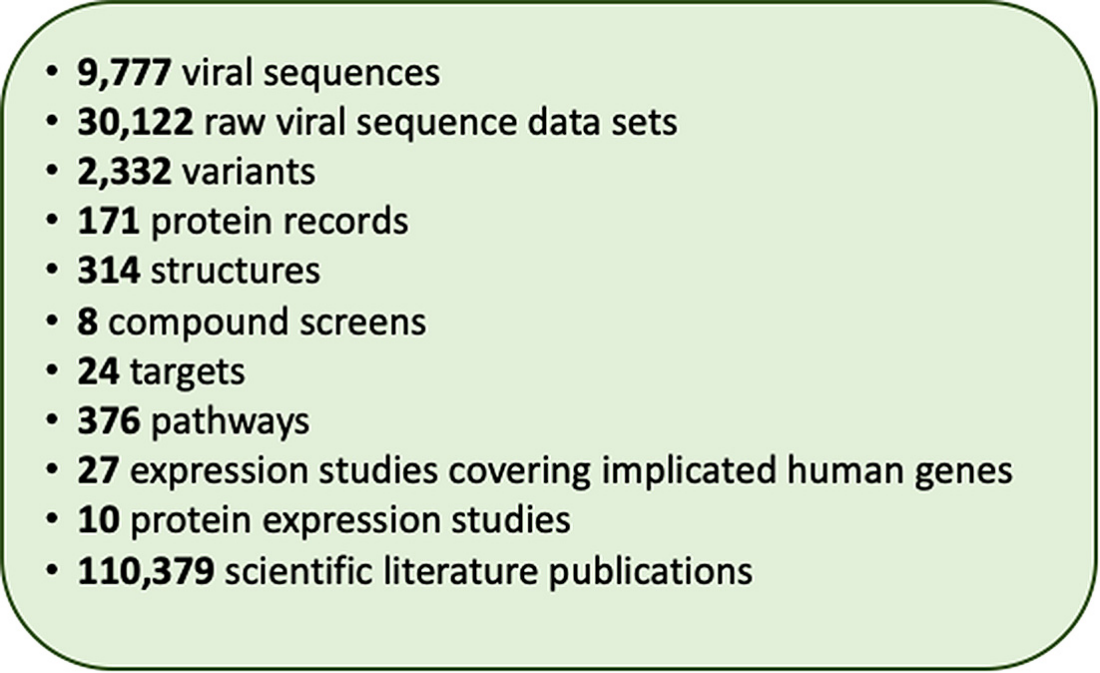

To integrate and make this data accessible to the user community and support research into COVID-19, several of the contributing data resources developed specific COVID-19 browsers and tools. For example, Ensembl has developed a SARS-CoV-2 genome browser and related resources (https://covid-19.ensembl.org/index.html), which support genome browsing on the sequence of the virus, its gene functions, variation data and information on its protein sequence (13). Providing reliable sequence information for COVID-19 is essential both for our understanding of the virus and its pathogenicity, and for the development of any vaccines or treatments. A further essential area of study is understanding the genetic factors that dictate susceptibility to the disease from Genome Wide Association Studies (GWAS). The GWAS Catalog aggregates data from large cohort studies, making curated SNP-trait associations and summary statistics from both genome-wide and targeted studies (18). In the past year, the GWAS Catalog has released a new data deposition service, recognising the need to make available data from preprints prior to peer-reviewed publication. At the time of writing, there were 3997 studies from pre-prints with summary statistics, 4 of which were COVID-19 related. There were 8 published studies and 25 available published associations between SNPs and COVID-19 susceptibility.

EMBL-EBI resources have also been able to support drug discovery research to the COVID-19 portal. ChEMBL, EMBL-EBI’s manually curated database of bioactive molecules with drug-like properties, has been curating the bioactivity data of potential anti-SARS-CoV-2 drugs. Drug discovery researchers have been screening candidate compounds to identify those with potential anti-SARS-CoV-2 activity, to identify already approved medications that can be repurposed for COVID-19. To date, several large-scale drug screening studies have been described and made available to the scientific community, either via preprints or as peer-reviewed publications. ChEMBL launched a special release in May 2020 (ChEMBL_27) focusing on studies that assess the potential anti-SARS-CoV-2 activity of compounds, with a further focus on studies that use cell-based assays in their screens. To date, ChEMBL has curated eight datasets on anti-SARS-CoV-2 compounds. Across all studies, 142 compounds have been shown to have some anti-SARS-CoV-2 activity (however, some of these also showed a high degree of cytotoxicity alongside their antiviral activity). Interestingly, only a few compounds have so far been shown to be active against SARS-CoV-2 in multiple studies: 14 compounds have been shown to be active in more than one study, and only 5 have shown activity across three or more studies. The ChEMBL_27 release also enables users to view the COVID-19-related results alongside previously available information for each compound, and to compare results for the same compound from different research groups.

**Figure 4.**
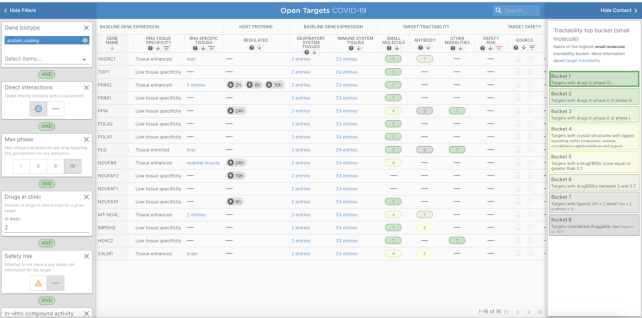
The Open Targets COVID-19 Target Prioritisation Tool. This example displays the results (listed in the centre window) of filters for targets that are: protein coding, have a direct interaction with a viral protein, have at least two drugs (for any indication) in a phase IV clinical trial, have a modulating compound in *in-vitro* assays, and have safety information (left content side bar). The right content sidebar displays further information for targets when entries in the list are selected. In this example, the small molecule target tractability button was selected for a particular target to display the level of predicted tractability information for the target.

EMBL-EBI is a partner in, and also hosts, Open Targets, a public-private partnership that uses human genetics and genomics data for systematic drug target identification and prioritisation (19). Open Targets has responded to the COVID-19 pandemic by developing a unique tool to aid filtering and prioritisation of human and viral (SARS-CoV-1 and SARS-CoV-2) proteins as potential drug targets for COVID-19 treatment (https://covid19.opentargets.org/). The tool integrates key datasets from publicly available resources via a simple user interface and is designed to filter potential drug targets by key properties of the data (Figure 4) to answer key questions such as ‘which human proteins directly interact with a SARS-CoV-2 viral protein, are expressed in the respiratory system, and have compounds with in vitro activity against COVID-19 that modulate this target?’.

Datasets integrated in the tool include:

- Protein interaction and network data on whether the target interacts directly with a SARS-CoV-1 or -2 viral protein, or with another virus protein (source: IntAct).
- Expression, distribution, tissue and subcellular location data for a given human target (source: Human Protein Atlas and Expression Atlas).
- Whether target abundance is up- or down-regulated during SARS-CoV-2 infection, and at which time points (20).
- Drugs in clinical trials that modulate the target, including details on the number of drugs and maximum trial phase, and whether these trials are for COVID-19 treatment (source: ChEMBL).
- Compounds that modulate the target and have been tested *in vitro* against SARS-CoV-2 and whether they had activity against the virus or not (source: ChEMBL).
- Target tractability assessment, including suitability for modulation with a small molecule or antibody (source: Open Targets Platform).
- Target safety information including known toxicity and organs affected (source: Open Targets Platform).
- List of peer-reviewed articles in which human targets co-occur with COVID-19 (source: Europe PMC).

The COVID-19 Data Portal also provides links to data submission systems, which provide data to the Platform, a bulk data download function, and links to currently 56 external data resources and services, spanning areas such as computational, clinical and epidemiological content. Ongoing work on the Portal includes the development of dashboard functions, integration of SARS-CoV-2 Data Hubs, a variety of data visualisations and deeper connections into clinical data (such as via a ‘Cohort Browser’).

The COVID-19 Data Portal also provides connections with other related resources, which at the time of writing included 5 other European projects, 7 computing support resources and 30 other databases and atlases containing information relevant to COVID-19 research. Examples of related resources and projects include the Johns Hopkins University COVID-19 Case Tracker (https://www.covidtracker.com/), Coronavirus Phylomes from Phylome DB (http://beta.phylomedb.org/covid19) and the European Clinical Research Infrastructure Network (ECRIN) COVID-19 Taskforce (https://ecrin.org/covid-19-taskforce).

### The SARS-CoV-2 Data Hubs

The SARS-CoV-2 Data Hubs are the second component of the European COVID-19 Data Platform. They focus on providing tools and services for the validation, processing, analysis, interpretation, sharing and publication of viral sequence data. The SARS-CoV-2 Data Hubs are deployed to be used by public health agencies and scientists responsible for generating viral sequences at national or regional levels, with substantial user and tool support to help with their use. Specific tools on offer at the time of writing include a dedicated raw viral data uploader, a number of cloud-based processing workflows supporting data from different sequencing platforms and library preparation methods and a Notebook-based data exploration and visualisation system. Ongoing work includes the addition of further workflows, including phylogenetic analysis. A particular focus has been placed on ensuring that COVID-19 Data Hubs promote FAIR Data Principles, ensuring data remains Findable, Accessible, Interoperable and Reusable (1).

At the time of writing, there were 16 national SARS-CoV-2 Data Hubs, supporting 70% of data. Viral data included over 420 000 data records, including 97 000 raw viral sequence data sets from 38 countries and over 330 institutions.

### The Federated European Genome-phenome Archive

The Federated European Genome-phenome Archive (FEGA) focuses on sensitive human biomolecular data relating to COVID-19 and is the third component of the European COVID-19 Data Platform. For these data, which are often required to remain within national borders, FEGA offers a unifying technology that allows national databases to be deployed and operated as nodes within a broader federation. This approach allows consistent interfaces (such as for submission and data access) across the federation while respecting requirements for national governance. Public metadata can be shared across the network so data discovery can operate from any point and lead to unambiguous endpoints for access requests and, ultimately, data access.

The COVID-19 pandemic has provided increased impetus for FEGA to become more widely operational to ensure rapid availability of COVID-19 host studies. This has led to an acceleration in the development of FEGA, including support for its underlying architecture, technical interfaces and software implementation to establish national nodes. To date, work has progressed on the governance and legal framework for FEGA, the technical interfaces, and demonstrator implementations in collaboration with the ELIXIR nodes (21). In addition, FEGA has been able to contribute to global standards for data sharing through the Global Alliance for Genomics and Health (GA4GH), a policy-framing and technical standards-setting organisation for responsible genomic data sharing. Its contributions have included standards for variation formats, secure data streaming, reference retrieval, data use ontology, encryption containers and phenotype exchange.

### Building the COVID-19 Data Platform

EMBL-EBI was uniquely positioned to rapidly deploy a COVID-19 Platform due to a number of factors. First, EMBL-EBI has a 25 year history in operating bioinformatics infrastructure to provide biomolecular data and services. This infrastructure includes systems for the at-scale rapid capture, integration and publication of scientific data from the research community. Specifically, several EMBL-EBI data resources for relevant data types were already in place and, in many cases, already holding data of direct relevance to SARS-CoV-2 and COVID-19 research (e.g. EGA, PRIDE, Ensembl). Second, drawing on over 600 staff expert in bioinformatics, software engineering and data science, we were able to rapidly redirect human and computational capacity towards the effort. Third, our work over the last six years with European partners in pathogen data systems through the EU Horizon 2020 COMPARE project, particularly around the COMPARE Data Hubs (22), has allowed us to develop specific extensions to our existing infrastructure. Fourth, as a part of ELIXIR, with strong connections to national nodes, we have well-established working relations with those who operate infrastructure in national health and public health systems and the relevant European-level Research Infrastructures that sit alongside ELIXIR. Finally, our commitment to open science, through open data, FAIR principles (1) and data standards, has facilitated the integration of the diversity of data types of relevance to COVID-19 research.

While the Platform continues to be developed very actively with the incremental addition of new data, functions and services, we count a number of key milestones to date:

- Rapid deployment of the three components of the European COVID-19 Data Platform—the SARS-CoV-2 Data Hubs, the Federated EGA and the COVID-19 Data Portal;
- Integration of SARS-CoV-2 and COVID-19 data from 14 EMBL-EBI Data Resources and connection into a number of external resources and services;
- Mobilisation of raw viral sequence data at scale: with almost no sharing of raw viral sequence data in March 2020, by September the Platform has mobilised some 25 000 data sets, with 80% of these data routed through the SARS-CoV-2 Data Hubs; raw data are essential for systematic variation and assembly analysis with appropriate error handling;
- Establishment of a host of new tools, including a viral data submission tool (https://ebi-ait.github.io/sars-cov2-data-upload/), a selection of viral data processing workflows and a SARS-CoV-2 genome browser (https://covid-19.ensembl.org/index.htm);
- Deep collaboration with a broad range of partners at scientific and infrastructure level, and creation of a network of national coordinators and Platform nodes, such as in Sweden, Japan, Norway, Poland, and Slovenia (https://covid19dataportal.se, https://covid19dataportal.jp, https://covid19dataportal.no, https://covidhub.psnc.pl, https://covid19dataportal.si).
- As of October 2020, the COVID-19 Data Portal had received over 2.9 million web requests, from over 90 000 unique hosts. Since the COVID-19 Data Portal is an open resource, it is difficult to estimate the exact number of users who accessed it. The number of unique hosts, while not an exact count, is used as a useful indicator of the number of users.

While the COVID-19 crisis has driven the specific implementation of the European COVID-19 Data Platform, we have, by design, built concepts and technologies that will be sustained long into the future. Drawing on the foundation of existing EMBL-EBI and ELIXIR Data Resources, all new data available through the Platform are appropriately routed towards their respective underlying data resources for long-term preservation. Using such applications as EMBL-EBI Search (3), the future discoverability of the underlying data is assured. The new software elements that we have developed are reusable not only for rapid deployment in future infectious disease scenarios, but also in the service of broader scientific research, such as genetic disease applications of FEGA. Finally, our network of partners in the Platform, the data standards that we have worked upon and the conventions and practices that we have established will enrich our opportunities for data infrastructure development in the future.

## SUPPORTING THE RESEARCH COMMUNITY THROUGH A GLOBAL CRISIS

Empowering cooperation in the specific field of coronavirus research is not the only way EMBL-EBI supports the research community. EMBL-EBI continues to support the life sciences research communities, both through its training programme and its regular service provision.

### Training

An essential part of the work at EMBL-EBI, alongside its data services and research activities, is its Training programme. The EMBL-EBI Training programme is now in its tenth year and aims to train scientists at all levels to get the most out of publicly available biological data (www.ebi.ac.uk/training). We responded rapidly to the COVID-19 pandemic; although we had to cancel some courses at short notice, we have created a robust mechanism for the delivery of highly interactive virtual courses and have received encouraging feedback from participants.

The training programme currently has five major components:

**Live virtual:** Aiming to provide the same impact as our face-to-face courses in a virtual environment, to reduce our carbon footprint, shield our participants from communicable disease and increase accessibility.
**Train anytime:** A comprehensive collection of online bioinformatics courses, pre-recorded webinars and course materials, allowing individuals to choose when, where and how they learn.
**Trainer support:** Enabling trainer communities to develop relevant and high-quality training courses, supported by EMBL-EBI experts and materials, and underpinned by global standards.
**Face-to-face:** Delivering courses to develop practical skills and knowledge, at EMBL-EBI and at host organisations worldwide (face-to-face training currently suspended).
**Visits and secondments:** Visiting scientists are embedded in an EMBL-EBI group for up to 6 months, often leading to longer-term collaboration (visits and secondments are currently suspended).

This combination of activities allows us to deliver training that is both of high quality and scalable: in 2019, we participated in 305 training and outreach events, and we delivered three train-the-trainer events, training 26 bioinformatics instructors. Meanwhile, in 2019 our ‘train online’ collection was accessed by 599 549 unique IP addresses and we expanded our online training offerings by adding two new courses and 59 new webinars. The expansion of our virtual training content in 2019 allowed us to respond promptly and effectively to the challenges of 2020, when we were able to adapt our programme to the restrictions brought on by the COVID-19 pandemic.

Furthermore, in late 2019 and early 2020 we began testing the use of commercial cloud-based systems for their potential to provide on-demand compute capacity for computationally intensive off-site courses. We can now create virtual machines (VMs) and Docker containers in the commercial cloud, support trainers to test their software and then provide access to the final VM/container/set of containers for trainees, giving them a very similar user experience to the experience that they would have if they were being trained face-to-face at EMBL-EBI. We accelerated our first use of this system in response to the COVID-19 pandemic.

### Empowering access through service provision

Crucially, EMBL-EBI empowers international cooperation through its regular service activity, enabling searchers from across the globe to collaborate daily by sharing their data through EMBL-EBI data resources.

All EMBL-EBI resources have been continuing their service development capabilities through the past year. Significant progress has been achieved in a number of core EMBL-EBI resources, including many referenced above (7,9,13) as well as Pfam (23), Rfam (24), and RNACentral (25).

We have been working to support the research communities that rely on our resources though both the COVID-19 pandemic and the ever-changing requirements of research. For example, the MetaboLights team (26) has been engaged with the metabolomic community in a comprehensive exercise to redevelop its submission systems to capture the growing amount and diversity of metabolomic information. By redeveloping the MetaboLights submission processes, we have enabled our user community to make more comprehensive and high-quality data available as easily as possible. At the time of writing, the database hosts around 700 publicly available studies with more in preparation and awaiting public release due to publication requirements. The MetaboLights team is committed to continually developing our resources and services to become a central hub for metabolomics related data and tools in collaboration with international collaborators and our diverse user community.

Data submission to other deposition databases has also changed through the recent COVID-19 pandemic. For instance, three of the months during the first half of 2020 have seen a record number of submitted data sets in a single month to the PRIDE database (8), the world-leading proteomics data repository and part of the ProteomeXchange Consortium (27), reaching 566 data sets (∼25 datasets per working day) submitted in June 2020 alone. The total number of proteomics data sets in PRIDE is currently around 18 000 and the number of proteomics datasets submitted to PRIDE continues to grow very significantly year by year. At the time of writing, 30 public data sets in PRIDE were COVID-19 related.

One of the strengths of EMBL-EBI as an institute is that it hosts services, training and research programmes within the same institute. Proteomics has been at the centre not only of expanded efforts by our services, but also of coronavirus-related research by our teams. Researchers from EMBL-EBI, in collaboration with partners from across the globe, have used proteomics to identify the mechanisms through which SARS-CoV-2 hijacks human cells during infection to promote its own replication. In turn these discoveries have allowed researchers to identify a number of existing drugs that may be effective at targeting the virus (28). Similarly, an international research group including scientists from EMBL-EBI identified hundreds of interactions between SARS-CoV-2 proteins and human proteins, which can in turn be used to identify potential COVID-19 treatments (29).

The activities of PRIDE are increasingly focused on integrating proteomics information into other added-value EMBL-EBI resources. In this context, more than 40 human and mouse quantitative proteomics datasets (both baseline and differential) are now available via Expression Atlas. Additionally, there is an ongoing effort to improve the annotation (and then facilitate the re-use) of public proteomics datasets by adopting and extending the file format SDRF (Sample and Data Relationship Format) used by the EMBL-EBI resources ArrayExpress and Expression Atlas (30).

As well as facilitating submission and access to its existing resources, EMBL-EBI is continually developing its resource portfolio to keep up with the ever-changing nature of molecular biology data. To this end, in 2020 EMBL-EBI has launched the eQTL Catalogue, a compendium of uniformly processed human gene expression data and splicing quantitative trait loci (31). The eQTL Catalogue focuses on expression of genetic variants that are associated with specific splicing events within genes, or with expression levels of nearby genes. eQTL datasets are rare, but they are hugely valuable for the process of drug discovery: data from the eQTL Catalogue has already been incorporated in the Open Targets platform for drug target identification and prioritisation.

## CONCLUSIONS

International cooperation, resource sharing and central data brokering are all principles upon which EMBl-EBI was founded. The COVID-19 pandemic has highlighted how important these practices are, and how crucial towards tackling and effectively addressing a global health crisis. International cooperation drove the creation and implementation of the European COVID-19 Data Platform, which in turn has connected scientists and practicing healthcare professionals not only throughout Europe, but all over the world. Cooperative resources have enabled new, ground-breaking research into the structure and mechanisms of action of the virus, which in turn are essential for the development of new effective treatments and vaccines.
